# Optimising Sensor Placement in Heritage Buildings: A Comparison of Model-Based and Data-Driven Approaches

**DOI:** 10.3390/s25134212

**Published:** 2025-07-06

**Authors:** Estefanía Chaves, Alberto Barontini, Nuno Mendes, Víctor Compán

**Affiliations:** 1 Advanced Production and Intelligent Systems Associated Laboratory, Institute for Sustainability and Innovation in Structural Engineering, Department of Civil Engineering, University of Minho, 4800-058 Guimarães, Portugal; 2 Department of Engineering and Geology, University “G. d’Annunzio” of Chieti-Pescara, 65127 Pescara, Italy; 3 Department of Building Structures and Geotechnical Engineering, University of Seville, 41012 Seville, Spain

**Keywords:** Optimal Sensor Placement (OSP), Structural Health Monitoring (SHM), heritage buildings, data-driven methods, experimental modal analysis (OMA), model uncertainty, dynamic identification, historical structures, sensor optimisation, heuristic methods

## Abstract

The long-term preservation of heritage structures relies on effective Structural Health Monitoring (SHM) systems, where sensor placement is key to ensuring early damage detection and guiding conservation efforts. Optimal Sensor Placement (OSP) methods offer a systematic framework to identify efficient sensor configurations, yet their application in historical buildings remains limited. Typically, OSP is driven by numerical models; however, in the context of heritage structures, these models are often affected by substantial uncertainties due to irregular geometries, heterogeneous materials, and unknown boundary conditions. In this scenario, data-driven approaches become particularly attractive as they eliminate the need for potentially unreliable models by relying directly on experimentally identified dynamic properties. This study investigates how the choice of input data influences OSP outcomes, using the Church of Santa Ana in Seville, Spain, as a representative case. Three data sources are considered: an uncalibrated numerical model, a calibrated model, and a data-driven set of modal parameters. Several OSP methods are implemented and systematically compared. The results underscore the decisive impact of the input data on the optimisation process. Although calibrated models may improve certain modal parameters, they do not necessarily translate into better sensor configurations. This highlights the potential of data-driven strategies to enhance the robustness and applicability of SHM systems in the complex and uncertain context of heritage buildings.

## 1. Introduction

The long-term preservation of heritage structures would strongly benefit from the implementation of effective Structural Health Monitoring (SHM) systems, capable of detecting damage at early stages and supporting informed conservation decisions [1]. Within SHM, the strategic selection and positioning of sensors plays a crucial role in maximising the relevance and quantity of acquired structural information, while accounting for practical and economic constraints on the number of sensors deployed. Optimal Sensor Placement (OSP) methodologies provide a systematic framework to address this challenge, identifying sensor configurations that best capture the dynamic behaviour of a structure.

Although OSP techniques have been extensively developed and applied in the context of modern civil infrastructure [2,3,4,5], their adoption in heritage buildings remains limited [6,7,8,9,10,11]. This gap is particularly significant given the distinctive characteristics of historic structures, including irregular geometries, heterogeneous materials, construction anomalies, and uncertain or evolving boundary conditions [12]. A recent state-of-the-art review [8] revealed that only a small number of studies directly addressed these challenges, with even fewer tailoring OSP strategies to the specific demands of heritage contexts.

Traditionally, OSP has been driven by numerical models that predict the modal properties and dynamic responses of the structure [13]. These model-based approaches, however, face notable challenges when applied to heritage buildings, where assumptions related to geometry, material properties, or boundary conditions often fail to reflect the actual structural behaviour. As a result, discrepancies between predicted and observed responses are common, particularly in structures affected by undocumented modifications or anomalies. To mitigate these issues, several contributions in the literature have sought to incorporate modelling and measurement uncertainties into the optimisation process [14,15,16,17,18,19] or have employed calibration techniques informed by experimental data [11]. Indeed, especially in the field of historical buildings, model calibration has emerged as a robust approach to enhance the reliability of the numerical models addressing the significant uncertainties related to the limited knowledge of the buildings structural and material characteristics [20,21,22].

Focusing on the second approach, based on model calibration, it is useful to distinguish between uncalibrated and calibrated model-based strategies, which are best understood as subsequent and complementary stages within the structural assessment process. The uncalibrated model, typically used in preliminary analyses, allows the fast simulation of various loading conditions and hypothetical scenarios without the need for prior measurements. This makes it a flexible and cost-effective tool in the early stages. However, it relies heavily on assumptions and may fail to replicate the actual behaviour of the building. In contrast, a calibrated model incorporates dynamic experimental data to enhance its representativeness, offering more accurate predictions and greater robustness for sensor optimisation [10]. Several approaches to calibration can be found in the literature, ranging from expert-guided manual adjustment, which allows for a more controlled tuning of specific structural parameters based on engineering judgement, to automated strategies such as Bayesian updating [23], genetic algorithms [20], or other model updating techniques, which improve reproducibility and reduce the subjectivity of the process. Nevertheless, developing a calibrated model is resource-intensive and demands technical expertise, namely high-quality experimental data and extensive characterisation efforts to achieve acceptable reliability [24,25]. Moreover, an accurate calibration may prove particularly challenging in complex or poorly documented structures, even with significant resources allocation.

As an alternative to model-based approaches, data-driven OSP has emerged as a promising methodology [26,27]. Although still less common in the literature, especially in the context of heritage buildings [28], this technique offers several notable advantages. Based solely on experimental data, it captures the real dynamic behaviour of the structure under actual operating conditions. This ensures that optimisation results are directly applicable to the current state of the building, avoiding the modelling uncertainties associated with assumptions on materials or boundary conditions. Additionally, the method relies on real sensor locations that have already been tested during the data acquisition process, ensuring the feasibility of these points and allowing the optimisation to account for practical considerations such as accessibility and sensor installation constraints.

However, the data-driven approach is not without limitations. Since it relies exclusively on the available measurements, it requires a carefully planned initial campaign to ensure sufficient spatial coverage of the structure. This may lead to the need for a large number of measurement points, increasing the cost and duration of the experimental phase. In contrast, model-based approaches, when calibration is used, can work with fewer measurement points, since they use the model to extrapolate behaviour across the structure. Furthermore, the data-driven method does not allow for the simulation of hypothetical scenarios such as extreme loading conditions or progressive damage states. While this is a key advantage of the model-based approach, it is important to highlight that such simulations are only meaningful if the model is truly representative of the real structure. Lastly, the quality of the experimental data is critical in data-driven OSP, as environmental noise or operational variability can significantly affect the results. This factor also affects the data used for model calibration, which means the robustness of both strategies can be influenced by similar experimental uncertainties.

In summary, each approach has specific strengths and limitations, and the choice between them depends on the objectives of the monitoring campaign, the availability of resources, and the degree of uncertainty that can be tolerated. Uncalibrated models offer fast and inexpensive analysis tools but rely on strong assumptions that may not hold in complex cases. Calibrated models strike a better balance between realism and predictive capability, though they require significant effort in development and calibration, which may not always succeed in capturing the true behaviour of the structure. Data-driven methods, on the other hand, provide results grounded in actual behaviour, avoiding modelling assumptions, but they can be limited in terms of scope and flexibility, and may require extensive experimental campaigns.

This study aims to contribute to the existing gap in the literature regarding the application of OSP methods to heritage structures, which often involve complex dynamic behaviour and significant contributions from local modes. These aspects are frequently overlooked in OSP studies, which typically focus on assets such as bridges, dams, and tall buildings. To this end, a real case study, representative of a common typology of religious architecture in southern Spain and reflecting key characteristics of a broader class of historical churches, was selected. The study also investigates the effectiveness of data-driven approaches, which remain underutilised in OSP applications but offer notable advantages over traditional model-based strategies, particularly in the context of the high uncertainty associated with heritage structures. A comparative evaluation is performed using three data sources: the uncalibrated model, the calibrated model, and experimental data. Several established optimisation criteria are employed to assess the impact of the chosen data source and metric. The analysis aims to deepen understanding of the viability of OSP in heritage buildings, the implications of data selection, and the relative performance of different optimisation metrics. The proposed methodology is designed to be systematic, transparent, and adaptable, providing a replicable framework applicable to other asset typologies.

This paper is organised as follows. Section 2 details the methodology employed. Section 3 describes the case study, including the building characterisation, the experimental identification campaign, and the development and calibration of the numerical model. Section 4 presents the OSP implementation and compares the results obtained from the different data sources, along with a discussion of their implications. Finally, Section 5 outlines the conclusions and potential directions for future research.

## 2. Methodology

This study investigates how the choice of input data affects OSP by comparing results obtained from three different sources of structural information: a numerical model in its initial uncalibrated state (FEM NOCAL), the same model after calibration using experimental data (FEM CAL), and purely experimental data obtained from an Operational Modal Analysis (OMA) campaign.

**Figure 1:**
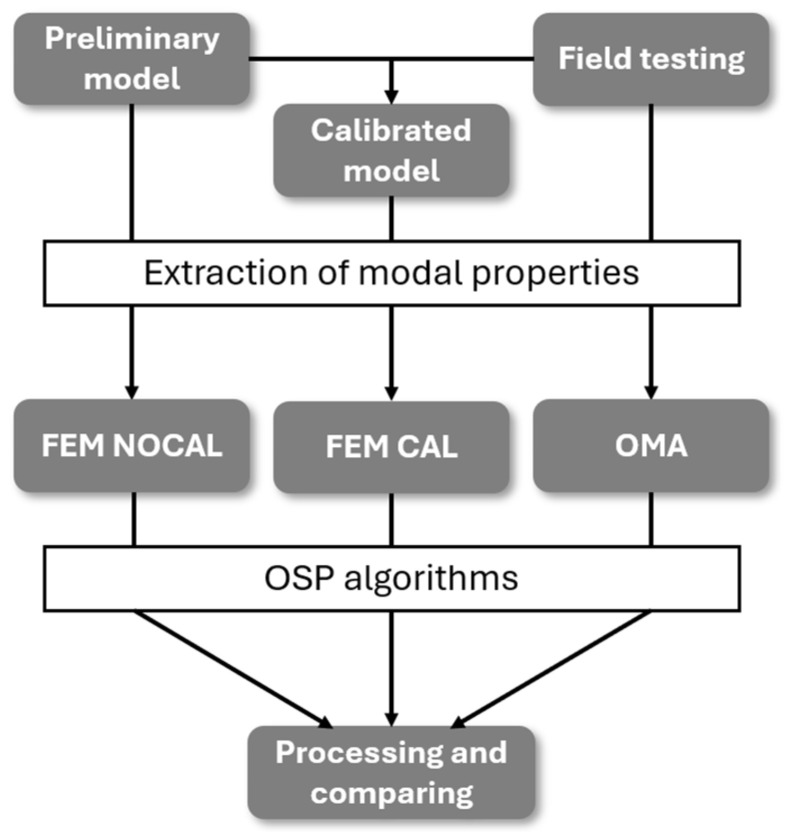
Methodological workflow.

The full methodological workflow is summarised in Figure 1, which outlines the key steps in the process. First, a preliminary numerical model of the structure is created using Abaqus CAE [29], incorporating geometric and material assumptions based on the available documentation, a visual inspection and basic Non-Destructive Test (NDT) data. Additionally, an experimental dynamic identification campaign is conducted, consisting of in situ measurements under ambient vibrations. The modal properties (natural frequencies and mode shapes) are extracted from this experimental dataset using the ARTeMIS Modal Pro 8.0 software [30].

The information obtained from the experimental campaign is then used to calibrate the preliminary FEM, resulting in the FEM CAL version. It is important to note that the calibration process makes full use of the complete set of data collected during the experimental campaign, including all identified modes and their corresponding frequencies and shapes, which are also the base for the data-driven optimisation.

These three datasets (FEM NOCAL, FEM CAL, and OMA) are subsequently used as input to the sensor placement process. For each of them, the same set of OSP algorithms is implemented, enabling a consistent comparative analysis of how the source of information affects the resulting sensor configurations.

A total of eight sensor placement algorithms are applied, covering a broad range of metrics and methodological approaches. Each algorithm quantifies different aspects of the modal information captured by each sensor or configuration, guiding the selection of sensors that maximise the relevance and quality of the recorded data, according to the specific optimisation metric. These algorithms can be classified into two main categories according to the nature of the metric they rely on. While the metric defines what is being optimised (e.g., energy contribution, linear independence, information content), the optimisation method determines how the sensor configuration is selected. In most cases, the type of metric constrains the optimisation approach, as some of them allow direct ranking while others require an evaluation of the complete sensor set.

The first group includes algorithms that assign a numerical value to each sensor location independently. This approach allows for a direct ranking of candidate locations based on their individual performance according to the selected metric, and the sensors with the highest scores are chosen to form the final configuration. Algorithms in this group include the Eigenvalue Vector Product (EVP) [31], Mode Shape Summation Plot (MSSP) [32], Average Driving Point Residue (ADPR) and Weighted ADPR (WADPR) [33]. These are energy-based metrics, favouring sensor positions where the dynamic response is more significant in terms of modal energy content. The EVP and MSSP algorithms aim to place sensors at locations where the target mode shapes exhibit the highest contributions, by maximising either their product (EVP) or their summation (MSSP). At the same time, these strategies avoid the selection of near-nodal points where modal displacements are close to zero. Similarly, the ADPR and WADPR metrics also seek to maximise modal displacements across the target modes weighted by their angular frequencies to favour lower modes.

The second group includes algorithms that evaluate sensor configurations as a whole, rather than assessing locations individually. In these methods, the value of the objective function depends on the collective contribution of the selected sensor set, typically seeking to ensure that the measured modes are as linearly independent as possible. This group comprises the Effective Independence (EfI) method [34], Estimation Error Minimisation (EEM) [35], Singular Value Decomposition ratio (SVDr) [36] and Modal Assurance Criterion off-diagonal terms minimisation (minMAC) [36]. In this case, the EfI method aims to maximise the independence of modal vectors measured at the selected sensors, while the EEM minimises the uncertainty in the reconstructed mode shapes. The SVDr promotes configurations that preserve the most significant components of the modal space and the minMAC criterion seeks to reduce redundancy by minimising correlations between the measured mode shapes, thus avoiding sensor placements that yield similar modal information.

The mathematical expressions of these objective functions are summarised in Table 1, where Φ denotes the modal matrix (for EEM Φ representing the full set of candidates and $\Phi_{m}$ considering just the partition to the measured locations), Λ the vector of natural frequencies, and $\sigma_{max}$ and $\sigma_{min}$ the largest and smallest singular value of Φ.

In terms of implementation, the optimisation process varies between the two groups. For the first group, the process is relatively simple: a single calculation provides a ranking of sensor locations, and the top N sensors are selected. In contrast, the second group requires a more computationally intensive approach, as the value of the objective function depends on the sensor configuration as a whole. Ideally, an exhaustive combinatorial search, commonly referred to as brute force (BF), would be used to identify the optimal configuration. However, due to the exponential increase in computational cost with the number of candidates, such approaches are rarely feasible in practice. Therefore, this study adopts a heuristic strategy: the Backward Sensor Sequential Placement (BSSP) method [13], an iterative elimination procedure where the least contributing sensor is removed at each step until the desired number of sensors remains. Although the BSSP method does not guarantee the global optimum, it provides an adequate trade-off between solution quality and computational efficiency. Since the results obtained in this study demonstrate a good performance of heuristic strategies, more complex metaheuristic methods, despite their potential advantage, were not considered necessary due to their higher computational cost and implementation complexity.

Any optimisation process requires the prior definition of a series of input parameters, which typically include the modal data, the set of target modes, the candidate sensor locations, and the number of sensors to be placed. These inputs shape the problem space and determine the nature of the resulting sensor configurations. Given the complexity of this type of problem and the lack of fast, standardised methods for evaluating large volumes of data, it becomes necessary to delimit the scope of the analysis to ensure the feasibility of the study.

To enable a systematic comparison, this study maintains all input parameters fixed across the different scenarios, with the only exception of the data source. This allows for an isolated evaluation of the influence of the information origin on the sensor selection outcome, removing other variables from the analysis. The values adopted for the remaining parameters, namely the set of target modes, the distribution of candidate locations, and the selected number of sensors, are tailored to the specific characteristics of the case study. Aiming at a comparative analysis, the target mode shapes are selected to include a representative combination of global and local responses to investigate the sensitivity of the metrics to distinct types of targets. Moreover, the number of sensors is defined according to the quantity and nature of the target modes. A small set, still larger than the number of modes, is preferred in this study to accentuate the differences between optimised configurations, since an excessively large number may lead to convergence and reduced sensitivity to the optimisation criteria. Therefore, six target modes, identified during the experimental dynamic campaign, are selected and a fixed number of eight sensors is defined accordingly. Further details on the definition of these parameters are provided in Section 4, following the thorough characterisation of the modal properties in Section 3.

The methodology adopted for the comparative analysis is primarily based on a qualitative approach. The sensor configurations obtained from each data source are represented graphically, highlighting coincident sensor locations across the different scenarios. This visual representation enables a direct comparison and supports an intuitive assessment of the degree of similarity between the configurations.

In addition to this overall visual comparison, special attention is paid to the distribution of sensors according to macroelements and directional orientation. This additional layer of analysis provides insight into whether, beyond exact sensor coincidences, the configurations tend to converge at a functional or structural level, that is, whether they prioritise similar structural components or directions of motion. This approach is particularly relevant considering the nature of the target modes selected for the analysis, which involve vibrations across different macroelements and spatial directions. Therefore, identifying convergence at this level supports a more nuanced evaluation of the consistency and robustness of the sensor placement strategies across different data sources.

In addition to the qualitative analysis, a quantitative evaluation is conducted to complement the comparison of results. For this purpose, the optimisation metrics are used as performance metrics (PM), enabling a more systematic assessment of the differences between scenarios. In this context, a performance metric quantifies how well a given sensor configuration satisfies the optimisation criterion when applied to a reference dataset. This approach allows the use of existing optimisation metrics as objective indicators to evaluate the quality of each solution relative to a known benchmark. The methodology involves computing the PM values for each scenario using a common reference modal matrix. These values are subsequently normalised with respect to the optimal result obtained for each metric across all scenarios considered. The normalised value of the PM associated with the optimisation criterion is then compared across scenarios, allowing a consistent interpretation of how variations in input parameters affect the quality of the solution. This approach provides a clearer understanding of the impact of each variable while maintaining a unified basis for comparison. This dual use of the optimisation metrics enhances interpretability by linking sensor configuration quality directly to the physical meaning of each criterion.

To complement the individual PM-based analysis, an aggregate indicator, the Performance Metric Index (PMI), is also introduced. The PMI is defined as the average of the normalised PM values associated with each configuration, considering all optimisation metrics as performance indicators. This formulation provides a balanced and comprehensive measure of overall performance, allowing for direct cross-comparison not only between different data sources but also across optimisation criteria. By integrating the results from multiple metrics into a single index, the PMI helps identify sensor configurations that yield more favourable outcomes in general terms, offering a global perspective on the robustness and effectiveness of each strategy.

## 3. Case Study

The Church of Santa Ana, located in Seville (Spain), was selected as the case study to support the application and validation of the methodology proposed in this work. Due to its architectural characteristics and construction typology, the building offers a representative example of historical religious structures commonly found in southern Spain, making it especially relevant for this study. Similar churches are widespread across Andalusia, sharing comparable features in terms of geometry, materials, and traditional construction techniques, as documented in previous studies [37]. This contributes to the broader relevance of the case, beyond the specific structure analysed here. For a detailed description of the recent investigations conducted on the Church, the interested reader is referred to [38].

### 3.1. The Church of Santa Ana

**Figure 2:**
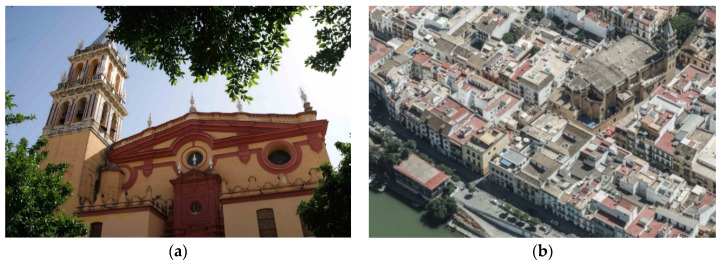
Santa Ana Church: (**a**) main façade; (**b**) aerial view of the church: rear façade and location with respect to the river.

The Church of Santa Ana (Figure 2), located in the Triana district of Seville, holds a unique place in the city’s history as the first Christian religious building constructed entirely from the ground up following the Reconquista in 1248. Its strategic position on the west bank of the Guadalquivir River, outside the city walls, also conferred upon it a defensive character, responding to the ongoing conflicts in the area during the early Christian occupation [39]. Commissioned during the reign of Alfonso X, the church exemplifies an architectural fusion of Gothic forms introduced from Castile and surviving Almohad influences, giving rise to an early Mudéjar style. Over time, the building has undergone a series of modifications, both restorative and expansionary, which have contributed to its complex and layered architectural identity [40].

Santa Ana follows a rectangular floor plan with three naves of five bays and a single apse. The central nave, taller and wider than the lateral ones, is covered by ribbed vaults and is supported by a regular grid of columns and pointed arches. Above the arches lies a gallery integrated within the wall thickness. Due to the defensive nature of the structure, the roof is nearly flat and walkable, finished with solid brick and later modified with a concrete slab whose details remain undocumented. The building spans approximately 37 m in length and includes distinctive features such as a tower (originally crenellated) and a crypt beneath one of the lateral naves [37].

The evolution of the church’s geometry reflects continuous adaptation, with chapels added progressively between the 15th and 19th centuries. These additions transformed the original layout into a more intricate and layered structure [39]. Furthermore, the church has suffered significant structural damage over the centuries due to seismic events. One of the earliest recorded earthquakes, in 1356, caused damage that required the reconstruction of the arches adjacent to the apse. During the devastating 1755 Lisbon earthquake, the vault above the choir collapsed and the main portal had to be entirely rebuilt [41].

The materials used in the original construction of the Church of Santa Ana reflect the region’s constraints and practices at the time. Brick, being more readily available and cost-effective, served as the primary construction material for most of the structures with lime-based mortar joints. Stone, on the other hand, was scarce and expensive, reserved for elements requiring greater strength or precision, such as arches, vault ribs, small columns, portals, and the base of the tower. The stone used is predominantly calcarenite, likely sourced from the Sierra de San Cristóbal [37].

From the in situ survey, three primary masonry typologies were identified and characterised through visual inspection using the Masonry Quality Index (MQI) methodology [42,43]. These include the vertical masonry of columns and walls, the brick vaults of the naves, and the hybrid masonry of the altar vaults. The MQI values allow an estimation of the mechanical property [44], summarised in Table 2.

To further assess the material properties, sonic tests [45,46] were conducted at twelve locations, using both direct and indirect transmission configurations [47], further details are provided in [38].

The results, presented in Table 3, highlight the differences between the two measurement points on each longitudinal wall (wall 1 and wall 2). The brick masonry near the main portal shows higher values, around 2 GPa, while the stone masonry reaches values above 3 GPa. Additionally, it is evident that the direct and indirect tests on the columns provide different results, with the indirect tests yielding lower values that are closer to those obtained for the brick masonry walls.

### 3.2. Dynamic Identification Campaign

**Figure 3:**
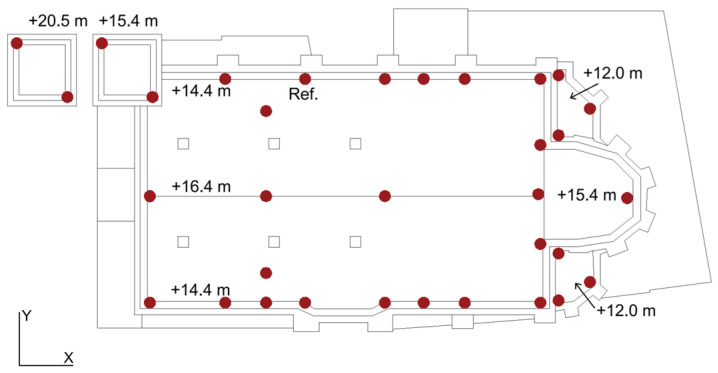
Location in plan of the 33 instrumented points, candidate nodes for the OSP.

An ambient vibration test was carried out to achieve the dynamic identification of the church. Figure 3 shows the 33 instrumented points that were strategically placed throughout the structure, on the nave’s roof (elevation +14.4 m to +16.4 m), on the lateral apse (+12.0 m), on the central apse (+15.4 m), and at two levels of the bell tower (+15.4 m and +20.5 m). Due to access limitations, the highest level of the tower was not included.

The test used five Kinemetrics EST force-balance triaxial accelerometers (bandwidth 0.01–200 Hz, dynamic range 155 dB, sensitivity 10 V/g). The campaign was divided into nine setups to cover all points, with one accelerometer fixed as a reference (REF) and the others moved between positions. Data were recorded using a 36-channel Obsidiana 36x system at 200 Hz over 20 min intervals per setup.

**Figure 4:**
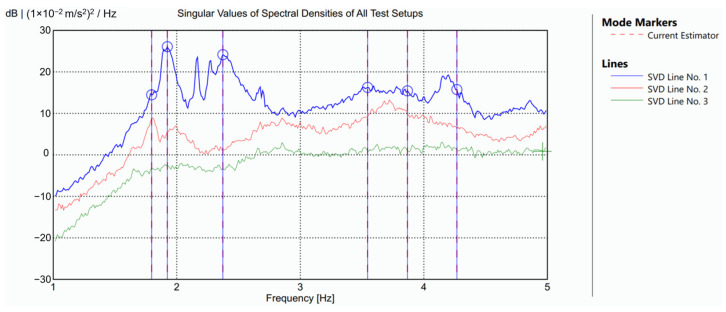
Experimental identification by the FDD method: singular values plot. Blue lines and circles indicating the selected modes.

Data processing was performed using ARTeMIS 8.0 software [30]. The identification process proved demanding, as not all dynamic responses produced clearly isolated spectral peaks. Figure 4 shows the singular value spectrum obtained from the global configuration using the Frequency Domain Decomposition (FDD) method, which guided the interpretation of the modal content.

**Figure 5:**
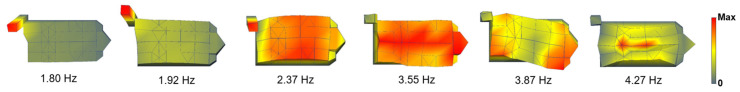
Identified modal shapes.

The identified modal shapes are illustrated in Figure 5. The first two modes are local vibration modes of the tower, with close frequencies of 1.80 Hz and 1.92 Hz. Both correspond to bending modes of the tower along one of its diagonals. The lower-frequency mode follows the diagonal formed by two free-standing corners and does not produce movement in the nave. Conversely, the higher-frequency mode involves the diagonal connecting one free corner with the corner adjoining the nave, in which case the tower’s motion induces a noticeable response in the nave.

The third mode, identified at 2.37 Hz, is a global mode involving transverse bending of the nave coupled with a bending motion of the tower, nearly parallel to the connecting face, and occurring in counterphase with the nave’s movement. The fourth mode, at 3.55 Hz, represents a longitudinal vibration of the nave with minimal tower involvement. The fifth mode, observed at 3.87 Hz, is a torsional mode of the nave. In both this torsional mode and the third mode, the longitudinal side of the nave not connected to the tower displays the highest vibration amplitudes. Finally, the sixth mode, identified at 4.27 Hz, corresponds to a local vertical mode concentrated in the roof structure of the nave.

### 3.3. Modelling

The development of the finite element model (FEM) began with the geometric reconstruction of the structure. This was achieved through the integration of existing drawings, in situ measurements, and detailed data extracted from a photogrammetric survey [38]. The use of photogrammetry proved essential to accurately represent the most complex architectural components, particularly the vaults of the nave and the altars. The level of geometric detail was chosen to provide a realistic representation of the main structural elements while omitting certain architectural features considered nonessential for structural behaviour.

Although this preliminary model provides a solid basis for understanding the global response of the structure, it involves a series of assumptions and simplifications that inevitably introduce uncertainties, especially regarding elements with limited accessibility or documentation. These limitations underline one of the key challenges in model-based approaches for OSP assessment.

Among the elements used to define the geometry of the nave are the perimeter walls, arches, and interior columns. These components presented a relatively low degree of uncertainty due to their straightforward geometries, ease of access, and the availability of visual inspection and non-destructive testing data to inform their material characterisation. In contrast, the roof structure was defined with a higher level of uncertainty. Although its geometry was externally captured through photogrammetry, the internal configuration, including cross-sections and constructive infill, was based on historical and technical literature [48,49,50], given the absence of direct access. Based on the span and rise of the vaults, a thickness of 0.2 m was assigned to the lateral vaults and 0.3 m to the central vault, each with a constructive infill equal to half the vault height. Additionally, a non-structural infill was modelled between the intrados of the vaults and the external roof plane, consisting of a concrete slab mentioned in documents from a 1970 intervention. However, no technical specifications of this slab were found, nor could this information be verified through alternative sources. The slab was estimated to be 10 cm thick and was modelled as being connected to the internal faces of the perimeter walls and the upper faces of the internal arches. The poor infill was modelled as a solid element connected to the slab and vaults, but disconnected from the vertical elements by a geometric setback of 1 cm. The vaults in the altar area were modelled following the same approach. Thus, the greatest source of uncertainty in the model lies in the assumptions made for the roof system, both in terms of geometry and material properties.

The tower was modelled based on original plans and direct measurements taken during the field campaign, including its vertical walls, two internal slab levels, and the pyramidal spire, which was represented using surface elements. Initially, the tower was assumed to be perfectly connected to the nave. Likewise, the vertical walls of the side chapels were modelled as monolithically connected to the nave. The foundation system was also included in the model and was assumed to extend to a depth of one metre below ground level, as no specific documentation or soil characterisation studies were available.

**Figure 6:**
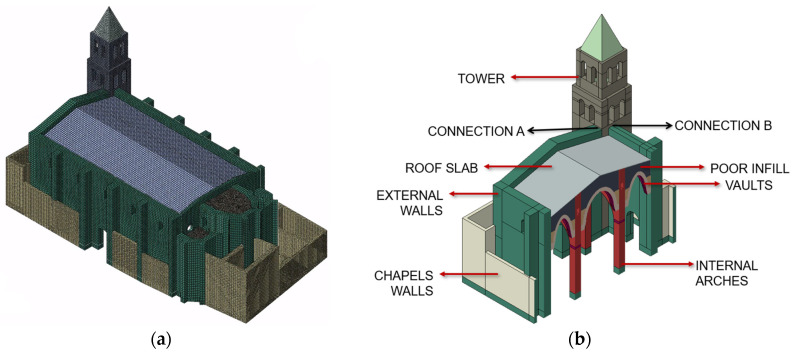
Numerical model of Santa Ana church: (**a**) FEM; (**b**) elements used as variables for the calibration.

The model was developed using Abaqus 6.14 [29]. After creating the geometry in a CAD environment, it was imported into the FEM software for meshing, using solid cubic elements. The final mesh comprised a total of 417,950 elements and 637,232 nodes, Figure 6a.

Regarding the definition of the material properties, five types were defined based on visual inspection and considering the results obtained from the MQI and sonic tests. The linear properties of these materials are specified in Table 4, namely the specific weight (W), the normal Young modulus (E), and the Poisson ratio (ν).

### 3.4. Calibration of the Model

The calibration process involved modifying selected properties of the finite element model to minimise discrepancies between the numerical and experimental modal responses. A preliminary sensitivity analysis was conducted considering variations of ±25% for fourteen parameters, individually, including Young’s modulus of brick masonry elements in various parts of the structure: the tower, nave walls, columns, arches, altar walls, vaults, structural and non-structural infills, and chapels. The list also included the stone masonry used for the ribs and capitals, and the concrete slab over the vaults. Additionally, the stiffness of the connections between the nave and the tower, and between the chapels and the nave, was also introduced as a variable. Following this analysis, some parameters were grouped to simplify the calibration process, see Figure 6b. This decision was based on engineering judgement, since the parameters had similar influence on the dynamic response and typically varied in a correlated manner during sensitivity analysis. For instance, the columns and arches were treated as a single material group, as were the nave and altar walls. The parameters related to the stone masonry were excluded from the calibration due to their negligible influence on the global dynamic behaviour of the structure.

Once the variables were identified through the sensitivity analysis, a calibration process was undertaken based on a manual iterative adjustment. This manual approach was adopted given the prior in-depth knowledge of the structure and the aim of gaining further insight into the influence of specific parameters on the dynamic behaviour. However, for practical applications or structures with greater uncertainty, the use of automated calibration techniques may improve efficiency.

**Figure 7:**
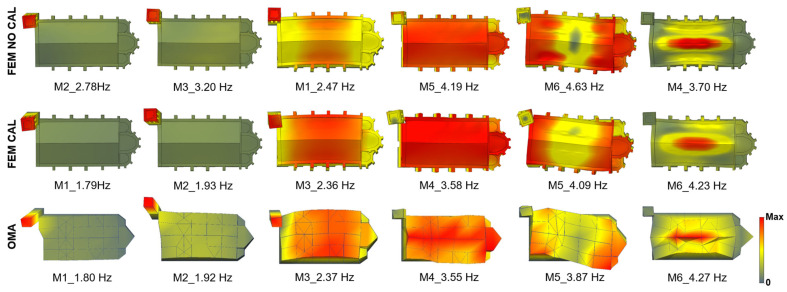
Dynamic properties comparison of the experimental (OMA) and numerical models (FEM), initial and calibrated. Chapels of the numerical model hidden for a clearer visual comparison.

In the initial stage of the calibration, the numerical model was able to capture the same global modal characteristics as the experimental results, although the order of the modes did not coincide and the frequency values presented notable discrepancies, as illustrated in Figure 7. These mismatches primarily affected the modes associated with the tower and the vertical mode of the nave roof.

For the tower modes, lowering the frequency values was necessary, which was achieved by reducing the stiffness of the tower–nave connection, thereby increasing the flexibility of the tower in the model. To reflect this in Abaqus, a surface-to-surface contact interaction was implemented between the tower and the nave. In the normal direction, a linear pressure–overclosure curve was defined, using the contact stiffness as a variable parameter during the calibration. For the tangential behaviour, a rough contact condition was applied to restrict any relative sliding between the parts. This adjustment enabled the correct sequencing of the first three modes and improved the frequency alignment of the two main tower-related modes. However, the third mode was the most sensitive to this connection, particularly due to its characteristic out-of-phase movement between the tower and the nave, which could not be replicated in the initial model, where both components moved in the same direction. For the fourth and fifth modes, the mechanical properties of the vertical masonry elements became the dominant factor in refining the frequency values.

For the sixth mode, the primary contributors were the concrete slab and the non-structural infill above the vaults. As previously noted, these elements involve a high degree of uncertainty due to limited available information. Their corresponding Young’s moduli were used as calibration parameters, and the tuning process led to a substantial increase in stiffness, especially for the weak infill layer, which in turn raised the frequency of this mode. This frequency increase caused a shift in the original fourth mode, which eventually appeared as the sixth, thereby improving the alignment and ordering of the final three modes. The final values adopted for each variable after calibration are presented in Table 5.

The alignment between the calibrated numerical modal properties and the experimental results is qualitatively illustrated in Figure 7 through the comparison of their respective mode shapes. However, considering the objectives of this study, it is essential to analyse the results in greater detail to understand the real differences among the three reference models that will serve as a basis for the optimisation process. For this purpose, the evaluation includes both the relative error of the natural frequencies and the Modal Assurance Criterion (MAC).

Table 6 presents the frequencies of the three models along with the associated errors computed with respect to the experimental data. In the case of the preliminary model (FEM NOCAL), significant discrepancies can be observed. The frequency errors vary widely and, in some cases, reach notably high values, resulting in a different modal sequence compared to the experimental reference. In contrast, the calibrated model exhibits a much closer match, with the exception of mode 5, all updated frequencies show errors below 1%, indicating a high level of agreement. Although mode 5 improves significantly (from 4.63 Hz to 4.09 Hz), it remains above the experimental reference of 3.87 Hz. This particular mode is influenced by numerous parameters, and further adjustments to improve its accuracy would have compromised the fitting of other modes. Consequently, the calibration prioritised the overall consistency of the remaining modes, as long as mode 5 remained within an acceptable range.

To complement this analysis, a broader perspective was adopted by examining not only the MAC value of each mode individually, but also the cross-MAC matrices comparing the OMA results with those of the two FEM (Table 7). In the uncalibrated model, the modal order was rearranged so that the diagonal elements of the matrix would align with the corresponding experimental modes. Even so, low MAC values are observed for the first three modes, while the last three show higher values. By contrast, the calibrated model demonstrates a stronger overall agreement with the experimental results: MAC values improve notably for the lower modes, as reflected in the diagonal values and the reduction in the off-diagonal terms. However, the last two modes show lower MAC values. This can be attributed to the increased complexity of higher-frequency modes, which tend to be more challenging to extract and interpret in the experimental analysis. Despite this, their visual correspondence with the numerical mode shapes supports their validity in the final model.

An important consideration arises from the behaviour of the uncalibrated model. The presence of relatively high non-diagonal MAC values, combined with the incorrect mode ordering, underscores a major limitation, since in the absence of reference data, such as modal frequencies or mode shapes, there is a substantial risk of misidentifying the target modes. This could lead to the selection of modes outside the expected frequency range or of limited structural relevance, thus significantly compromising the reliability of the subsequent optimisation process.

## 4. OSP Implementation

Based on the dynamic behaviour of the structure, the input variables for the optimisation were defined in line with the premises and objectives of the proposed methodology. The initial approach considered the complete set of six identified modes (M1-M6) as input data. Candidate sensor locations were limited to the 33 measurement nodes (99 DOFs) used during the experimental campaign, as shown in Figure 3.

Regarding the definition of the number of sensors, theoretical criteria establish that the number of measured degrees of freedom should be equal to or greater than the number of modes intended for identification [51], which in this case is six. Nevertheless, taking into account the geometric complexity of the structure and the variability of modal shapes involved, the number was increased to eight. This selection is considered sufficiently detailed to allow for meaningful observations across different configurations, while remaining manageable in terms of computational and interpretative effort. This number offers enough flexibility to explore the influence of sensor location on the quality of the results and supports a more robust evaluation of the proposed metrics and methodology.

### 4.1. Comparison

**Figure 8:**
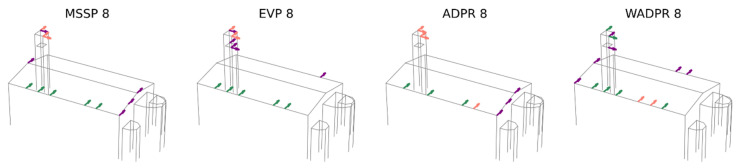

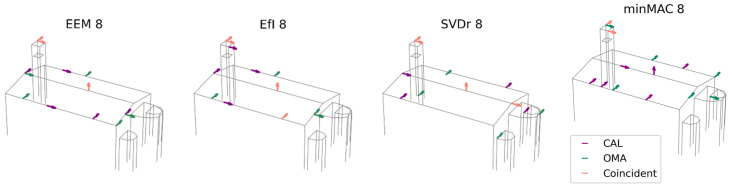
Result comparison: OMA vs. calibrated FEM data source; six target modes and eight sensors.

The first comparison involves the modal shapes obtained from the experimental campaign (OMA) and the calibrated finite element model (FEM CAL), which are expected to yield similar results. In this initial analysis, the six identified modes are targeted to optimise the placement of eight uniaxial sensors. The obtained results are presented in Figure 8.

The number of exact coincidences is relatively low. On average, 3.25 sensors coincide, with a range of 2 to 5 sensors depending on the optimisation technique used. Examining the individual responses of the metrics, ADPR exhibits the highest number of coincidences, with five shared sensors. However, four of them are located at the top of the tower, with only one in the nave. At the other extreme, the WADPR and minMAC metrics show the lowest number of coincidences, with only two sensors coinciding, in the first case in the nave and in the second case at the top of the tower. Coincidences in the nave are rather limited, with WADPR, EfI, and SVDr being the metrics that present the highest number of coincidences, with only two sensors in common in this area.

A more detailed analysis of the distribution of non-coincident sensors reveals that, in most cases, they do not follow similar distribution patterns either. These results are summarised in Table 8, where in addition to the number of exact coincidences, the distribution of sensors according to macroelement and direction is presented. This allows for a more direct comparison, with shaded cells indicating when the number of sensors of the same type coincides in the two scenarios compared. The classification is based on the macroelement, tower (T), nave (N), apse (A), and the direction, i.e., longitudinal (X), transversal (Y), and vertical (Z). Furthermore, a distinction has been made between the top (t) and lower (l) levels of the tower, as the latter is particularly significant in FEM-based results.

Only the ADPR and EEM metrics manage to maintain some consistency in sensor placement. The optimisation based on the calibrated FEM tends to prioritise the placement of a sensor in the vertical direction at the centre of the roof, as well as the placement of sensors at the lower level of the tower in some cases. Additionally, there is a tendency to locate transversal sensors on the transverse walls of the nave rather than in more central areas for the energy-based metrics, as observed in the OMA results.

However, both approaches share the same issue with energy-based metrics, since they exclude vertical and longitudinal sensors in the nave, sensors that may help in the identification of the longitudinal and vertical modes of the nave. Moreover, the apse does not appear to play a relevant role in the solutions derived from both cases.

**Figure 9:**
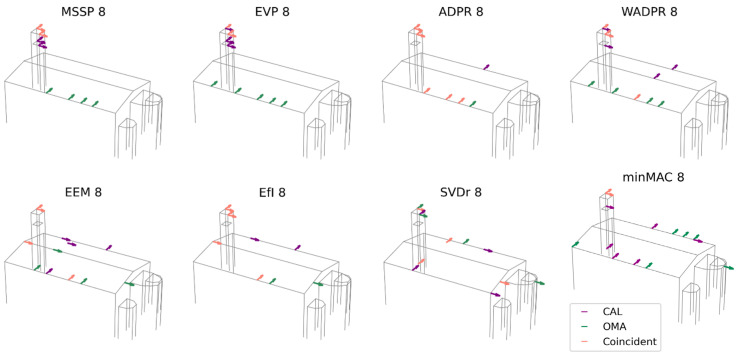
Result comparison: OMA vs. calibrated FEM data source; four target modes and eight sensors.

To further investigate whether the discrepancies observed are caused by the lower calibration accuracy of the two highest-frequency modes (Table 7, FEM CAL), an additional comparative analysis is performed. This time, the evaluation was limited to the first four modes (M1-M4), where a more precise calibration was achieved (MAC values between 0.80 and 0.97). The results of this comparison are shown in Figure 9.

The average number of coincident sensors increased to 4.25, with a broader range of coincidence spanning from 2 to 7 sensors. As in the previous case, most of these overlaps occur at the top of the tower, where up to four sensors coincide. However, in the nave, the number of matches varies between zero and three, showing a slight increase compared to the previous scenario. Among the analysed metrics, ADPR exhibits the highest number of coincidences, with seven out of eight sensors aligning, followed closely by EfI with six. Conversely, minMAC registers the lowest level of agreement, with only two coincident sensors in the tower, as in the previous scenario. Regarding the nave, ADPR and SVDr show the greatest alignment, with three coincident sensors.

For greater clarity, the results based on the distribution of sensors according to macroelement and direction are included in Table 9. If the placement of non-coincident sensors is also considered, it reveals that certain metrics, such as ADPR, EEM, and EfI, tend to preserve a more consistent distribution pattern. The results from the FEM approach reinforce previous observations, confirming that the apse remains an area of low relevance for sensor placement across all metrics. Furthermore, the FEM continues to prioritise sensor locations at the lower level of the tower, to an even greater extent than in the previous scenario. This can be attributed to the increased significance of the tower within the complete set of modes, as now half of the modes are local, and this element also plays a key role in the third mode. Meanwhile, the configuration based on OMA exhibits a greater concentration of NY sensors. Notably, the FEM calibrated results no longer favour NY sensors at the extremities of the nave, suggesting that this pattern may have been influenced by mode 5, which is now excluded from the analysis. A pattern emerges for the minMAC metric in the calibrated model, where sensors tend to be positioned in the half of the nave closest to the façade. Conversely, in the OMA case, they are primarily located in the opposite half, which corresponds to the altar.

**Figure 10:**
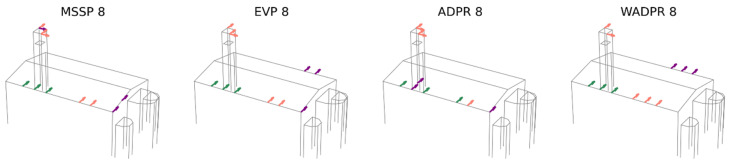

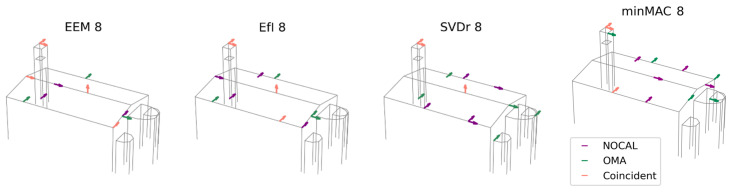
Result comparison: OMA vs. non-calibrated FEM data source; six target modes and eight sensors.

After analysing the results obtained from the comparison between OMA and the calibrated numerical model, a second set of comparisons is conducted using the modal shapes obtained from the vibration tests (OMA) and the uncalibrated numerical model (FEM NOCAL), considering all six target modes (M1-M6). This comparison is particularly relevant as it allows for an assessment of the influence of calibration on the optimisation results. The outcomes of this comparison are presented in Figure 10.

The number of matching sensor locations between OMA and the uncalibrated model ranges from 3 to 5, with an average of 4.38 coincident sensors, compared to 3.25 for the calibrated model. The coincidences are predominantly located in the upper part of the tower, where up to four sensors coincide, whereas in the nave, the number of matching sensors varies between 1 and 3.

Regarding the different metrics, several achieve the maximum number of coincidences (five sensors), including all energy-based metrics. Among the iterative metrics, MVM stands out. The metrics with the lowest number of coincidences are SVDr and minMAC, with three matching sensors, one in the nave and two at the top of the tower.

The analysis of the sensor distribution by macroelement and direction, detailed in Table 10, shows that the distribution pattern remains consistent for metrics such as EVP, ADPR, WADPR, EEM, and EfI.

The lower level of the tower is no longer considered relevant, with no sensors being selected in this region. This difference, in contrast to the calibrated model, explains the increased number of coincidences for several energy-based metrics, as the sensors previously positioned in the lower level of the tower in the calibrated model are now placed in locations that coincide with OMA. This shift could be attributed to the calibration of the connection between the tower and the nave, which in the calibrated model is significantly more flexible. This alteration was essential during calibration to ensure the order of the modes and the adjustment of the frequency values. Nonetheless, from the perspective of the sensor placement results, it appears to penalise the reliability of the model.

**Figure 11:**
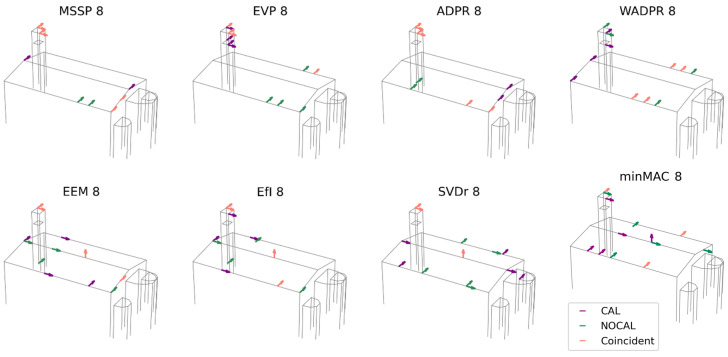
Result comparison: calibrated FEM vs. non-calibrated FEM data source; six target modes and eight sensors.

Building on the previous analyses, the results from both the calibrated and uncalibrated models are compared to evaluate the patterns proposed in each case and examine the coincidences and discrepancies between the obtained results. This comparison allows for a precise determination of the extent to which the calibration has influenced the results, offering a more detailed understanding of the impact of calibration on the optimisation process. The results are presented in Figure 11.

On average, the number of matching sensor locations between the two models is 4.25, ranging from 3 to 6 sensors. Coincidences primarily occur in the upper part of the tower, where up to four sensors coincide. In the nave, the number of matching sensors ranges between one and four, with both NY and NZ sensors contributing to the observed matches.

Regarding the different metrics, MSSP and ADPR exhibit the highest number of coincidences, with four sensors in the upper part of the tower and two in the nave. In contrast, SVDr, and the minMAC metrics present the lowest number of coincidences, with only three matching sensors in each case. Among the metrics, focusing on the nave, WADPR stands out with four coincident NY sensors.

The analysis of the distribution patterns (Table 11) shows that MSSP, ADPR, and EEM maintain a consistent matching pattern between both models. Additionally, if an AY sensor is considered equivalent to an NY sensor, SVDr can also be included in this group. As previously noted, the main difference between the two models lies in the sensor distribution within the tower. In the calibrated model, several metrics consider sensor placement in the lower level of the tower, whereas in the uncalibrated model, all sensors are located in the upper level. Despite this, the overall sensor distribution remains quite similar, both in the nave and in the apse, which is largely ignored in the sensor placement process, with just three sensors being positioned in this area.

### 4.2. Performance Metrics

In addition to the qualitative analysis presented above, a quantitative evaluation is carried out to complement the comparison of the results obtained from different data sources (OMA, FEM CAL, and FEM NOCAL). For this purpose, the optimisation metrics are used as performance metrics (PMs), allowing for a more systematic assessment of the differences between scenarios.

The modal matrix obtained from the OMA is adopted as a reference, as it is considered to best represent the real behaviour of the structure, considering also that it is the one the models aim to replicate. Therefore, all PMs are calculated by partitioning the OMA modal shape matrix to the final sensor set proposed by each algorithm. These values are then normalised based on the optimal value achieved for each PM across the three scenarios and the eight optimisation metrics. Although the direct comparison of PM values across scenarios is not entirely neutral (since all values are calculated with respect to the OMA matrix) this approach enables a clearer understanding of how the model-based results deviate from those considered closer to reality. In this way, a quantitative dimension is added to the previous analysis, contributing to more robust conclusions.

Table 12 presents the normalised values of the corresponding performance metric (for EFI, the determinant of the FIM, detFIM, is considered). The table also includes the associated error between the model-based results and the OMA reference. The results show, first, that in some cases the optimisation does not lead to the best outcome for the specific metric it aims to optimise in the OMA scenario. This means that, in these cases, an alternative algorithm, meant to optimise a different objective function, leads to a better value of the intrinsic metric of these methods. The discrepancy is observed for SVDr and minMAC. However, the deviation is only 3% of the best value. In the first case, the optimal value is achieved by the EEM proposal, while for the minMAC is achieved by the SVDr one. This confirms the fact that the SSP methods are inherently suboptimal.

As expected, the OMA based optimisation results are better than those obtained based on the numerical models. Furthermore, the results are consistent with the conclusions drawn from the qualitative analysis. The non-calibrated model results are generally closer to the OMA reference than those of the calibrated model, except in the case of SVDr, for which the calibrated model yields a higher number of matching sensors. This can be clearly observed in Table 12, where a red colour scale has been applied to the cells indicating the percentage error with respect to the highest OMA value, with darker shades corresponding to higher errors.

In general, the average error values are relatively high, with 44% for the calibrated model and 25% for the uncalibrated one. A clearer picture emerges when considering the nature of each metric, whether based on energy content or sensor set configuration. Among the energy-based metrics, ADPR and MSSP show the lowest error values in the calibrated model. In the case of ADPR, this outcome is consistent with both a high number of coincident sensors and a preserved distribution pattern. MSSP presents a more nuanced case. Although it does not maintain the expected sensor distribution and includes a lower number of matches, it still yields a low error, particularly for the calibrated scenario. This suggests a lower sensitivity of the metric to variations in the sensor location. In contrast, EVP and WADPR, despite showing similar levels of coincidence and preserving the spatial pattern in the uncalibrated model, result in substantially higher error values. This discrepancy highlights the importance of PM evaluation, as qualitative assessments alone may not fully explain the observed performance.

The set-based metrics also reveal relevant differences. EEM outperforms EfI in the calibrated model despite presenting fewer matches. This outcome is attributed to a better alignment with the expected distribution. In the case of EfI, although the number of coinciding sensors is higher, the deviation from the intended pattern leads to a greater error. Interestingly, when the number of matching sensors is identical for both calibrated and uncalibrated cases, the error remains significantly lower in the calibrated scenario, reflecting greater similarity of the pattern distribution.

SVDr further illustrates this behaviour. For the calibrated model, it shows a low error, which can be explained by a similar distribution of sensors when the apse and nave are considered together. In contrast, this logic does not hold for the uncalibrated model, where this metric shows few coincidences and a different pattern and still maintains a low error value, although higher than in the calibrated case.

Lastly, minMAC consistently exhibits a high error in both models. This is linked to the limited number of matches and significant divergence in spatial distribution. The discrepancy is especially marked in the calibrated case, where relevant sensors such as NZ are missing. In the uncalibrated model, the error is slightly reduced due to a more coherent distribution pattern.

As a complementary analysis, the maximum variation observed for each PM across all scenarios and optimisation methods was also calculated. This value, denoted as Δmax and included in the last column of Table 12, helps to assess the sensitivity of each metric to changes in sensor configuration. In this case, the first column of the table refers to the PM (e.g., detFIM for EfI), unlike for the rest of the table where it represents the optimisation criterion. The results reveal that detFIM, EEM, and SVDr exhibit the highest Δmax values, indicating a greater sensitivity to changes in placement and thus a stronger capacity to distinguish between different sensor layouts. In contrast, MSSP and ADPR show the lowest Δmax values (0.29 and 0.39, respectively), suggesting that their evaluation remains more stable across different configurations.

In addition to the comparative assessment among data sources based on the individual PM values, a Performance Metric Index (PMI) is introduced to enable a cross-evaluation not only across scenarios but also across optimisation metrics. This index allows for the identification of sensor configurations that yield more favourable outcomes in overall terms, irrespective of the specific metric targeted by each optimisation. To ensure an unbiased formulation, the PMI is defined as the average of the normalised PM values associated with each configuration, considering all optimisation metrics employed as performance indicators. In this study, the PMI comprises four energy-based and four set-dependent metrics, thus providing an equilibrated and representative measure of global performance.

Table 13 presents the PMI values for all three cases, along with the relative error of the two model-based scenarios with respect to the results obtained using OMA. Globally, considering the aggregated results of all metrics, the OMA scenario yields the highest total PMI value, followed by the non-calibrated model, while the calibrated model obtains the lowest value. This difference between the two model-based scenarios is also reflected in the sum of relative errors, with the calibrated model showing a higher average error percentage.

When analysing the individual values for the PMI, it is observed that the highest values are obtained for the EfI metric in both the reference scenario (OMA) and the non-calibrated model. In contrast, for the calibrated model, the highest PMI value corresponds to SVDr. The SVDr exhibits the lowest relative error in the calibrated case, which aligns with the observations made in the analysis of performance metrics in the previous table. On the other hand, the lowest PMI values are generally recorded for the energy-based metrics, regardless of the scenario considered.

In terms of relative error with respect to the reference values, WADPR stands out as the metric with the highest error percentage for the calibrated model (43%). In the case of the non-calibrated model, the higher percentage is provided by the EEM metric (20%) but is very close to the other metrics.

### 4.3. Discussion

Comparing OMA and FEM CAL over six target modes, the number of exact coincidences is relatively low. Furthermore, the localisation patterns are not preserved, highlighting significant discrepancies between the two analysed scenarios. The highest agreement occurs in the tower, a macroelement with local modes and a limited number of candidates, which demonstrates greater stability in sensor selection. Here, up to four sensors overlap, whereas in the nave, the range is more limited, with a maximum of two coincident sensors in some cases. Additionally, energy-based metrics exhibit the same limitations in both approaches, lacking fundamental sensors for the identification of the complete set of modes (NX and NY).

It is important to emphasise the high variability observed in the results, even though the two data sources should, in principle, be closely aligned and represent the same structural scenario upon the calibration. This divergence can be attributed to both the inherent noise in the experimental data and the discrepancies that persist despite the model updating, particularly with regard to the higher modes. Although the calibration was exhaustive and yielded satisfactory results, the problem itself presents inherent limitations, as detailed in Section 3.4. As a result, optimisation does not fully converge between the two approaches considered.

By reducing the target modes to prioritise only those with higher agreement in the calibration process, the overall number of coincident sensors has increased. This improvement can be explained by two main factors. First, the reduced number of target modes maintaining the number of sensors decreases the variability in the optimisation results. Second, the increased relevance of the tower in the selected modes, particularly due to the relevance in the first two modes and its role in the third global mode, leads to a more consistent sensor allocation in this area. This tendency, combined with the improved calibration of the selected modes, helps explain the higher level of agreement observed in this case. Nevertheless, while greater consistency is achieved in several metrics, such as EfI, EEM, and ADPR, substantial differences still emerge in others, especially among the minMAC and energy-based metrics, indicating that significant discrepancies remain despite a more reliably calibrated mode set selection.

Considering the non-calibrated model, the results exhibit a counterintuitive consistency between the results obtained based on the experimental and numerical data. Contrary to expectations, the results from the uncalibrated model show greater overall similarity to the OMA results than the calibrated model. The unexpected result challenges common assumptions regarding the benefits of calibration in OSP strategies and motivates a closer examination of its practical implications, as discussed in the following analysis. For example, it may suggest that certain adjustments introduced during the calibration process may influence the optimisation in unintended ways.

However, it is important to note that, despite being a preliminary model, the uncalibrated version already captures the six target modes with modal shapes that are reasonably consistent with the experimental results. Although some cross-MAC values are not particularly high, the modal shapes are sufficiently similar to yield sensor locations that coincide with those obtained from OMA. Indeed, even for the modes with lower MAC values, the locations contributing most to the modal displacement are the same and only the overall shape presents discrepancies with the experimental one.

A clear example is the tower, a key structural element in the sensor distribution. In the uncalibrated model, the predominant amplitude characteristics of the tower are already present, even though the overall modal shapes are not perfectly aligned. This, combined with the limited number of candidate sensor locations available on this element, results in a high number of sensor coincidences with the experimental configuration.

On the other hand, while the calibrated model provides a more accurate representation of the overall modal shapes, it likely introduced localised discrepancies, as demonstrated again by the tower where certain metrics tend to place sensors at the lower level. This may be related to the complexity of calibrating the tower’s modal shapes, which depended heavily on the modelling of a flexible connection with the nave.

For an accurate interpretation of these results, it is essential to consider the specific context of the study, where complementary experimental data were available for a confident interpretation of the mode shapes of the non-calibrated model. In practical applications, such reference information is typically unavailable when working with preliminary models, which increases the risk of optimisation based on incorrect or unrealistic mode shapes. For example, significant differences in frequency ranges, as observed in this case, may not affect the optimisation itself, since it relies mainly on the modal matrix, but they can lead to divergences in the identification and selection of target modes.

It is also important to note that, although the uncalibrated model yields a higher number of sensor locations coinciding with those derived from OMA, on average one more than the calibrated model, this difference is not consistent across all metrics. While most metrics, especially energy-based ones, as well as EEM and minMAC, show a higher number of coincidences for the uncalibrated case, others remain unchanged (ADPR, EfI), and some display a reduced match (SVDr). When comparing the results of the calibrated and uncalibrated models, the number of matching sensor locations is higher. However, the average remains close to that obtained in the comparison with the OMA. While calibration clearly influences the optimisation results, minMAC and SVDr appear to be the most sensitive to these changes, yielding only three matching sensor positions.

To further understand these discrepancies, a quantitative analysis based on individual performance metrics (PMs) was carried out. These metrics allow for a more systematic evaluation of each sensor configuration’s performance relative to the OMA reference. The results confirm that the OMA scenario consistently yields the highest PM scores. However, the non-calibrated model often outperforms the calibrated one. Notably, metrics such as EEM and ADPR present lower errors in the non-calibrated case, while minMAC shows poor performance in both models, confirming its strong sensitivity to mismatches in spatial distribution. EfI, on the other hand, displays similar levels of sensor coincidence in both cases but a significantly lower error in the calibrated model, which is explained by the better spatial alignment in that configuration.

To provide a global measure of performance, the Performance Metric Index (PMI) was introduced. Defined as the average of the normalised PM values across all metrics, the PMI enables a direct comparison of sensor configurations. In terms of metric performance, set-dependent metrics (e.g., EfI, EEM) achieve the highest PMI values across scenarios, while energy-based metrics perform less consistently. This suggests that some criteria are more robust to variations in input data and better suited for complex structures.

These findings highlight the importance of analysing metric performance in relative rather than absolute terms, as each criterion responds differently to variations in the sensor layouts. Instead of identifying universally superior metrics, the discussion aims to elucidate how each one behaves under varying problem inputs and data sources, providing insights into their suitability and limitations in practice.

## 5. Conclusions

This paper has evaluated the impact of different data sources on the outcomes of Optimal Sensor Placement (OSP) strategies for heritage buildings. A methodology was developed to systematically compare sensor configurations obtained from three different sources of modal information: experimental data (OMA), a calibrated numerical model (FEM CAL), and a non-calibrated version of the same model (FEM NOCAL). The analysis was applied to a real case study, the Church of Santa Ana in Seville, Spain. Eight optimisation criteria were used to generate sensor layouts, which were then assessed through both qualitative and quantitative approaches. The qualitative analysis was based on direct visual comparison, focusing on coincident sensor locations and examining spatial distribution patterns across macroelements and directional axes. The quantitative analysis included the use of individual performance metrics (PMs), calculated with respect to a common reference, and a global indicator, the Performance Metric Index (PMI), which aggregates normalised results across all metrics.

The results highlight several key findings:

- The data source has a decisive influence on the optimisation results. Notable differences emerge in the sensor configurations proposed by the same criteria, particularly in areas with a greater number of candidates and larger variability in the contribution to the target mode shapes, such as the nave. In contrast, the tower shows greater stability across all cases, due to its local modal characteristics and limited number of candidate locations.
- Reducing the number of target modes increases the level of sensor coincidence, particularly when those modes are more accurately captured by the models. However, this improvement is not consistent across all metrics. While some criteria such as EfI, EEM, and ADPR show better agreement under this condition, others, including minMAC and certain energy-based metrics, remain sensitive to changes in the input data.
- The non-calibrated model shows an unexpected level of agreement with the experimental results. In several metrics, it performs better than the calibrated model, suggesting that certain calibration adjustments may introduce localised distortions in modal representation that negatively affect sensor placement optimisation.
- minMAC and SVDr criteria are the most sensitive to variations in the data source. In contrast, other metrics such as EfI and EEM present intermediate behaviour, maintaining a more stable optimised sensor pattern across scenarios while still reflecting differences in input data.
- PMs and PMI comparisons confirm the superiority of the OMA-based configurations and show that the non-calibrated model systematically achieves better overall performance than the calibrated one. Overall, set-dependent metrics yield higher PMI values, especially EfI and SVDr, whereas energy-based metrics generally result in poorer performance.
- The metrics EfI, EEM, and SVDr show higher variation (Δmax) in their values when used as performance metrics, indicating greater sensitivity to sensor placement. This behaviour reinforces their suitability for this role, as they better capture differences in the quality of the selected configurations.

In conclusion, by combining qualitative analysis with structured quantitative indicators such as PMs and PMI, a more robust and balanced framework for OSP assessment can be achieved, helping identify which configurations are not only optimal for a specific criterion but also more generally robust. Overall, the findings highlight the importance of both the selected metric and the data source in defining effective OSP strategies. The observed discrepancies underline the limitations of relying exclusively on numerical models, even when calibrated, and support the adoption of data-driven approaches in heritage structures, particularly in contexts where accurate numerical models are difficult to obtain.

Although the analysis focuses on a single case study, the Church of Santa Ana is considered representative of a broader set of historical buildings that share similar geometrical, material, and constructive characteristics, especially within the regional context of southern Spain. As such, the general trends identified here are expected to be applicable to comparable scenarios. Furthermore, the methodology proposed for evaluating sensor configurations, which combines multiple data sources and performance indicators, is adaptable to other typologies and offers a practical framework for guiding OSP decisions under high uncertainty conditions. Future developments could explore the implementation of the proposed workflow as a semi-automated tool for heritage monitoring applications.

Additionally, while this study is not based on deep-learning algorithms, future research could investigate how hybrid approaches such as transfer learning might support the generalisation of OSP strategies across buildings with similar typological characteristics.

## Figures and Tables

**Table 1:** OSP metrics objective functions.

MSSP	EVP
$f_{MSSP}\left(\Phi \right)=max\left(\sum_{j=1}^{N}\;\left|\Phi_{ij}\right|\right)$	$f_{EVP}(\Phi )=max\left(\prod_{j=1}^{N}\;\left|\Phi_{ij}\right|\right)$
**ADPR**	**WADPR**
$f_{ADPR}(\Phi )=max\left(ADPR\right)$ $ADPR_{j}=\frac{1}{n}\sum_{i=1}^{n}\;DPR_{ij}$	$f_{WDPR}(\Phi )=max\left({DPR}_{min}⊗ADPR\right)$ $DPR=\Phi_{ij}⊗\Phi_{ij}\Lambda^{-1}$
**EEM**	**EfI**
$f_{EEM}\left(\Phi ,\Phi_{m}\right)=min\;tr(\Gamma )$ $\Gamma =\Phi {\left(\Phi_{m}^{T}\Phi_{m}\right)}^{-1}\Phi^{T}$	$f_{EfI}(\Phi )=max\left(min\left(\mathrm{diag}\left(E\right)\right)\right)$ $E=\Phi {\left(\Phi^{T}\Phi \right)}^{-1}\Phi^{T}$
**SVDr**	**minMAC**
$f_{SVDr}(\Phi )=min\left(\frac{\sigma_{max}}{\sigma_{min}}\right)$	$f_{minMAC\;}(\Phi )=min\sqrt{\frac{1}{k(k-1)}\sum_{i\neq j}\;{MAC}_{ij}^{2}}$

**Table 2:** Average material properties of each type of masonry according to the MQI assessment.

	f_m_ [MPa]	τ_0_ [MPa]	E [MPa]	G [MPa]	W [kN/m^3^]
Vertical elements	4.26	0.078	1845	533	18
Nave vaults	4.84	0.099	2050	860	18
Altar vaults	6.00	0.148	3175	972	20

**Table 3:** Sonic tests outcomes.

Element	Type	Distance [m]	Material	Number of Joints	E [GPa]	Aver. [GPa]
Wall 1	indirect	0.76	brick	10	1.46	1.52
Wall 1	indirect	0.90	brick	10	0.85	
Wall 2	indirect	0.80	brick	10	1.82	
Wall 2	indirect	0.81	brick	10	1.03	
Column 2	indirect	0.80	brick	10	1.53	
Column 1	indirect	0.83	brick	10	1.63	
Main portal	indirect	0.88	brick	10	2.07	
Main portal	indirect	0.70	brick	10	1.75	
Column 1	direct	1.35	brick	–	2.72	2.54
Column 2	direct	1.35	brick	–	2.36	
Altar	indirect	1.35	stone	–	3.10	2.88
Altar	indirect	1.27	stone	–	2.65	

**Table 4:** Material properties of each type of masonry.

Material	Elements	W [kN/m^3^]	E [MPa]	ν
Masonry general	Ext. walls, int. columns, and arches, tower, chapels	18	1500	0.2
Masonry vaults	Vaults and structural infill	18	1500	0.2
Stone masonry	Ribs, capitals	20	2900	0.2
Poor infill	Poor infill	0.9	25	0.2
Concrete	Roof slabs	22	25,000	0.2

**Table 5:** Variables of the FEM calibration process. Initial and updated values.

Element	Variable	Initial Value	Updated Value
Tower (Tw)	Youngs’s modulus [MPa]	1500	1150
Ext. walls	Youngs’s modulus [MPa]	1500	1150
Int. arches	Youngs’s modulus [MPa]	1500	1150
Vaults	Youngs’s modulus [MPa]	1500	1300
Poor infill	Youngs’s modulus [MPa]	25	110
Roof slab	Youngs’s modulus [MPa]	25,000	35,000
Chapels walls	Youngs’s modulus [MPa]	1500	1000
Tw-nave A	Connection [MPa m]	connected	14
Tw-nave B	Connection [MPa m]	connected	10
Tw-chapel A	Connection [MPa m]	connected	100
Tw-chapel B	Connection [MPa m]	connected	100

**Table 6:** Experimental and numerical frequencies.

Mode	OMA	FEM NOCAL		FEM CAL	
OMA	Frequency [Hz]	Frequency [Hz]	Frequency Error	Frequency [Hz]	Frequency Error
Mode 1	1.8	2.78	54.4%	1.79	−0.6%
Mode 2	1.92	3.02	57.3%	1.93	0.5%
Mode 3	2.37	2.47	4.2%	2.36	−0.4%
Mode 4	3.55	4.19	18.0%	3.58	0.8%
Mode 5	3.87	4.63	19.6%	4.09	5.7%
Mode 6	4.27	3.7	−13.3%	4.23	−0.9%
RANGE	1.8/4.27	2.47/4.63	−13.3/57.3	1.79/4.23	−0.9/5.7

**Table 7:** Cross-MAC matrix for OMA vs. FEM NOCAL and OMA vs. FEM CAL. The red scale is used to emphasize larger MAC values.

		FEM NOCAL								FEM CAL					
		M2	M3	M1	M5	M6	M4			M1	M2	M3	M4	M5	M6
**OMA**	M1	0.68	0.03	0.28	0.00	0.01	0.00	**OMA**	M1	0.92	0.02	0.04	0.00	0.00	0.00
	M2	0.01	0.33	0.64	0.00	0.00	0.00		M2	0.09	0.80	0.05	0.00	0.00	0.00
	M3	0.12	0.37	0.44	0.00	0.04	0.00		M3	0.00	0.00	0.97	0.00	0.08	0.01
	M4	0.05	0.08	0.00	0.89	0.01	0.01		M4	0.02	0.02	0.00	0.91	0.00	0.00
	M5	0.09	0.00	0.00	0.17	0.70	0.01		M5	0.03	0.01	0.02	0.21	0.61	0.06
	M6	0.01	0.01	0.00	0.00	0.00	0.72		M6	0.01	0.00	0.01	0.00	0.04	0.53

**Table 8:** Distribution of sensors according to macroelement and direction for the OMA and calibrated FEM data source taking six target modes and eight sensors into consideration. Cells shaded in grey indicate agreement of results between the two scenarios.

Metric	Coincid.	Source	TXt	TYt	TXl	TYl	NX	NY	NZ	AX	AY
MSSP	3	OMA	2	1	0	0	0	5	0	0	0
		FEM CAL	2	2	0	0	0	4	0	0	0
EVP	3	OMA	2	1	0	0	0	5	0	0	0
		FEM CAL	2	2	1	2	0	1	0	0	0
ADPR	5	OMA	2	2	0	0	0	4	0	0	0
		FEM CAL	2	2	0	0	0	4	0	0	0
WADPR	2	OMA	1	1	0	0	0	6	0	0	0
		FEM CAL	0	1	1	0	0	6	0	0	0
EEM	3	OMA	1	1	0	0	2	3	1	0	0
		FEM CAL	1	1	0	0	2	3	1	0	0
EfI	4	OMA	1	1	0	0	1	4	1	0	0
		FEM CAL	2	1	0	0	2	2	1	0	0
SVDr	4	OMA	1	1	0	0	1	2	1	0	2
		FEM CAL	1	1	0	0	2	2	1	0	1
minMAC	2	OMA	2	1	0	0	0	4	0	1	0
		FEM CAL	1	1	0	0	1	4	1	0	0

**Table 9:** Distribution of sensors according to macroelement and direction for the OMA and calibrated FEM data source taking four target modes and eight sensors into consideration. Cells shaded in grey indicate agreement of results between the two scenarios.

Metric	Coincid.	Source	TXt	TYt	TXl	TYl	NX	NY	NZ	AX	AY
MSSP	4	OMA	2	2	0	0	0	4	0	0	0
		FEM CAL	2	2	2	2	0	0	0	0	0
EVP	3	OMA	1	2	0	0	0	5	0	0	0
		FEM CAL	2	2	2	2	0	0	0	0	0
ADPR	7	OMA	2	2	0	0	0	4	0	0	0
		FEM CAL	2	2	0	0	0	4	0	0	0
WADPR	4	OMA	1	2	0	0	0	5	0	0	0
		FEM CAL	2	2	1	0	0	3	0	0	0
EEM	4	OMA	1	1	0	0	3	3	0	0	0
		FEM CAL	1	1	0	0	3	3	0	0	0
EfI	6	OMA	2	2	0	0	2	2	0	0	0
		FEM CAL	2	2	0	0	2	2	0	0	0
SVDr	4	OMA	2	1	0	0	1	3	0	1	0
		FEM CAL	1	1	0	0	3	3	0	0	0
minMAC	2	OMA	1	1	0	0	0	5	0	1	0
		FEM CAL	1	1	1	0	1	4	0	0	0

**Table 10:** Distribution of sensors according to macroelement and direction for the OMA and non-calibrated FEM data source taking six target modes and eight sensors into consideration. Cells shaded in grey indicate agreement of results between the two scenarios.

Metric	Coincid.	Source	TXt	TYt	TXl	TYl	NX	NY	NZ	AX	AY
MSSP	5	OMA	1	2	0	0	0	5	0	0	0
		NOCAL	2	2	0	0	0	4	0	0	0
EVP	5	OMA	1	2	0	0	0	5	0	0	0
		NOCAL	1	2	0	0	0	5	0	0	0
ADPR	5	OMA	2	2	0	0	0	4	0	0	0
		NOCAL	2	2	0	0	0	4	0	0	0
WADPR	5	OMA	1	1	0	0	0	6	0	0	0
		NOCAL	1	1	0	0	0	6	0	0	0
EEM	5	OMA	1	1	0	0	2	3	1	0	0
		NOCAL	1	1	0	0	2	3	1	0	0
EfI	4	OMA	1	1	0	0	1	4	1	0	0
		NOCAL	1	1	0	0	1	4	1	0	0
SVDr	3	OMA	1	1	0	0	1	2	1	0	2
		NOCAL	1	1	0	0	2	3	1	0	0
minMAC	3	OMA	2	1	0	0	0	4	0	1	0
		NOCAL	1	1	0	0	2	4	0	0	0

**Table 11:** Distribution of sensors according to macroelement and direction for the calibrated and non-calibrated data source taking six target modes and eight sensors into consideration. Cells shaded in grey indicate agreement of results between the two scenarios.

Metric	Coincid.	Source	TXt	TYt	TXl	TYl	NX	NY	NZ	AX	AY
MSSP	6	CAL	2	2	0	0	0	4	0	0	0
		NOCAL	2	2	0	0	0	4	0	0	0
EVP	4	CAL	2	2	1	2	0	1	0	0	0
		NOCAL	1	2	0	0	0	5	0	0	0
ADPR	6	CAL	2	2	0	0	0	4	0	0	0
		NOCAL	2	2	0	0	0	4	0	0	0
WADPR	4	CAL	0	1	1	0	0	6	0	0	0
		NOCAL	1	1	0	0	0	6	0	0	0
EEM	4	CAL	1	1	0	0	2	3	1	0	0
		NOCAL	1	1	0	0	2	3	1	0	0
EfI	4	CAL	2	1	0	0	2	2	1	0	0
		NOCAL	1	1	0	0	1	4	1	0	0
SVDr	3	CAL	1	1	0	0	2	2	1	0	1
		NOCAL	1	1	0	0	2	3	1	0	0
minMAC	3	CAL	1	1	0	0	1	4	1	0	0
		NOCAL	1	1	0	0	2	4	0	0	0

**Table 12:** Normalised PM values and comparison for the data source analysis. The red scale is used to emphasize larger errors.

	PM			Error vs. OMA		Δmax of Each PM
	OMA	CAL	NOCAL	CAL	NOCAL	
MSSP	1.00	0.85	0.94	15%	6%	0.29
EVP	1.00	0.39	0.68	61%	32%	0.84
ADPR	1.00	0.94	0.97	6%	3%	0.39
WADPR	1.00	0.33	0.64	67%	36%	0.86
EEM	1.00	0.64	0.84	36%	16%	0.98
EfI	1.00	0.18	0.67	82%	33%	1.00
SVDr	0.97	0.91	0.71	6%	27%	0.94
minMAC	0.97	0.19	0.52	80%	47%	0.88
Average	0.99	0.55	0.75	44%	25%	0.77

**Table 13:** PMI values and comparison for the data source analysis. The red scale is used to emphasize larger errors.

	PMI			Error vs. OMA	
	OMA	CAL	NOCAL	CAL	NOCAL
MSSP	0.52	0.36	0.42	31%	19%
EVP	0.52	0.37	0.45	29%	14%
ADPR	0.52	0.38	0.43	27%	18%
WADPR	0.5	0.28	0.42	43%	16%
EEM	0.69	0.47	0.55	31%	20%
EfI	0.74	0.48	0.61	35%	18%
SVDr	0.59	0.56	0.55	6%	8%
minMAC	0.65	0.54	0.58	16%	10%
Average	0.59	0.43	0.5	27%	15%

## Data Availability

The data are available on request.

## Abbreviations

The following abbreviations are used in this manuscript:

ADPR	Average Driving Point Residue
BSSP	Backward SSP
DOF	Degree of Freedom
DPR	Driving Point Residue
EEM	Estimation Error Minimisation
EfI	Effective Independence
EfIDPR	Effective Independence DPR
EVP	Eigenvalue Vector Product
FDD	Frequency Domain Decomposition
FEM	Finite Element Model
FEM CAL	FEM calibrated
FEM NOCAL	FEM non-calibrated
IEI	Information Entropy Index
MAC	Modal Assurance Criterion
minMAC	Minimum off-diagonal MAC
MSSP	Mode Shape Summation Plot
MVM	Modified Variance Method
NDT	Non-Destructive Testing
NODP	Non-optimal Driving Point
OMA	Operational Modal Analysis
OSP	Optimal Sensor Placement
PM	Performance Metric
PMI	Performance Metric Index
PSD	Power Spectral Densities
QRD	QR decomposition
SHM	Structural Health Monitoring
SSP	Sensor Sequential Placement
SVDr	Singular Value Decomposition ratio
VM	Variance Method
WDPR	Weighted ADPR
